# Missed opportunities for HIV testing in newly-HIV-diagnosed patients, a cross sectional study

**DOI:** 10.1186/1471-2334-13-200

**Published:** 2013-05-02

**Authors:** Karen Champenois, Anthony Cousien, Lise Cuzin, Stéphane Le Vu, Sylvie Deuffic-Burban, Emilie Lanoy, Karine Lacombe, Olivier Patey, Pascal Béchu, Marcel Calvez, Caroline Semaille, Yazdan Yazdanpanah

**Affiliations:** 1 ATIP-Avenir Inserm: “Modélisation, Aide à la Décision, et Coût-Efficacité en Maladies Infectieuses”, 152 rue du professeur Yersin, Loos 59120, France; 2 Université Lille Nord de France, Lille EA2694, France; 3 Service des Maladies Infectieuses, CHU de Toulouse, Toulouse, France; 4 Institut de Veille Sanitaire, Saint-Maurice, France; 5 Institut Gustave Roussy, Service de Biostatistique et Epidémiologie, Villejuif, France; 6 AP-HP Hôpital Saint-Antoine, Paris, France; 7 Inserm, UMR-S707, Paris, France; 8 Université Pierre et Marie Curie, Paris, France; 9 Service des Maladies Infectieuses et Tropicales, CHI Villeneuve-Saint-Georges, Villeneuve-Saint-Georges, France; 10 Inserm CIC9301, Lille, France; 11 CHRU de Lille, Lille, France; 12 Université Européenne de Bretagne, Rennes, France; 13 CNRS UMR6590, Rennes, France; 14 Service de maladies infectieuses et tropicales, AP-HP Hôpital Bichat Claude Bernard, Paris, France; 15 Université Denis Diderot, Paris, France

**Keywords:** HIV/AIDS, HIV testing, Late diagnosis, Risk assessment, Access to care

## Abstract

**Background:**

In France, 1/3 HIV-infected patients is diagnosed at an advanced stage of the disease. We describe missed opportunities for earlier HIV testing in newly-HIV-diagnosed patients.

**Methods:**

Cross sectional study. Adults living in France for ≥1 year, diagnosed with HIV-infection ≤6 months earlier, were included from 06/2009 to 10/2010. We collected information on patient characteristics at diagnosis, history of HIV testing, contacts with healthcare settings, and occurrence of HIV-related events 3 years prior to HIV diagnosis. During these 3 years, we assessed whether or not HIV testing had been proposed by the healthcare provider upon first contact in patients notifying that they were MSM or had HIV-related conditions.

**Results:**

1,008 newly HIV-diagnosed patients (mean age: 39 years; male: 79%; MSM: 53%; diagnosed with an AIDS-defining event: 16%). During the 3-year period prior to HIV diagnosis, 99% of participants had frequented a healthcare setting and 89% had seen a general practitioner at least once a year. During a contact with a healthcare setting, 91/191 MSM (48%) with no HIV-related conditions, said being MSM; 50 of these (55%) did not have any HIV test proposal. Only 21% (41/191) of overall MSM who visited a healthcare provider received a test proposal. Likewise, 299/364 patients (82%) who sought care for s had a missed opportunity for HIV testing.

**Conclusions:**

Under current screening policies, missed opportunities for HIV testing remain unacceptably high. This argues in favor of improving risk assessment, and HIV-related conditions recognition in all healthcare facilities.

## Background

In the past decade in developed countries, highly active antiretroviral therapies have dramatically decreased HIV infection morbidity and mortality [1-3]. When HIV care is initiated early, patient’s life expectancy becomes closer to that of the general population [4,5]. More recently, compelling evidence has demonstrated benefits of early treatment of HIV-infected patients for the global population by reducing HIV transmissions [6,7]. Consequently in some countries like US or France, treatment guidelines have moved toward initiating earlier HIV therapy, i.e. at CD4 cell counts <500/mm^3^[8-10]. However, most patients are diagnosed long after the optimal moment of treatment initiation. Recent studies estimated that approximately 30% of people living with HIV in Europe are unaware of their infection [11]. Other studies, mostly from western Europe, estimated that 24-39% of HIV-infected patients presented for care at an advance stage of the disease (with AIDS and/or CD4 cell count <200/mm^3^) [12-19]. Late diagnosis compromises benefits of antiretroviral therapies.

In most European countries, risk-factor-based HIV testing strategy alone shows limits to detect HIV-infected people because 1) people do not consider themselves at risk and 2) healthcare providers fail in risk assessment [20-24]. In line with the US CDC recommendations [25,26], in 2008 the UK guidelines stated that HIV screening should be considered in general practice and for all general medical admissions in regions where HIV prevalence exceeds two per 1,000 [27]. In 2009, new French guidelines recommended one-time routine voluntary HIV screening to be implemented population-wide in France [28,29]. However, these recommendations are not applied because of feasibility and budget impact issues [30-32].

Few studies have evaluated patient consultations during the possibly-HIV-infected period [22,33-36], and the bulk of them were carried out in the US or the UK. To assess the situation in France, the objectives of the present study conducted in newly diagnosed HIV-infected patients were to describe: 1) in all patients, frequenting of healthcare settings prior to HIV diagnosis during the period in which they were likely to be HIV-infected; and 2) in at-risk populations (MSM) and patients with possibly HIV-related conditions, opportunities for testing proposal and consequently earlier HIV diagnosis.

## Methods

### Study design, setting and population

A cross-sectional study was conducted between June 2009 and October 2010 in 69 ANRS centers providing care to patients with HIV throughout France (metropolitan France and French overseas departments). The study was proposed to all the ANRS centers (around 160) on a voluntary basis; 64 centers accepted to participate and enrolled effectively patients. The two mains reasons for not participating were lack of manpower and too few new HIV-infected patients per year. However, centers from smaller hospitals were recruited to ensure geographic coverage of French territories.

Patients were eligible for this study if they were over 18-years-old, had an initial HIV-positive test (diagnosis) in France, and sought care in a participating center within the 6-month period following HIV diagnosis. Patients diagnosed in a foreign country or who had been living for ≤1 year in France were excluded, since opportunities for testing were not in France. Eligible patients were consecutively enrolled during the study period.

The study received approval from two French data protection authorities (CCTIRS and CNIL) and did not require formal ethical approval according to French regulations. All patients received information and gave written consent before their inclusion in the study.

### Definition

We defined situations that would lead care providers to suggest HIV testing, i.e. to persons belonging to a high-risk group for HIV acquisition and who presented with HIV-related clinical conditions. Risk groups for acquiring HIV infection were (by descending risk) [37]: injected drug users (IDU), men who have sex with men (MSM), heterosexuals with at-risk behavior (≥2 sexual partners and/or unprotected sex with casual partners within the past three years) and immigrants from an HIV endemic country of Sub-Saharan Africa (born and living there until moving to France). For patients belonging to several risk groups, the group with the highest risk was retained.

Possibly HIV-related conditions included symptoms more frequent during chronic stage of HIV infection and diseases associated with a high HIV prevalence (>1%) [35,37-40]:

General symptoms: fever unexplained and/or lasting ≥1 month; diarrhea recurrent and/or lasting for ≥1 month; weight loss ≥10%;

Cutaneous/mucous symptoms: seborrheic dermatitis; oral herpes; oral hairy leukoplakia; oral candidiasis; varicella zoster; onychomycosis; unexplained prurigo. This class also included generalized lymphadenopathy;

Bacterial infections: community-acquired pneumonia, pulmonary tuberculosis, recurrent bacterial infections;

Diseases associated with high prevalence of HIV infection: viral hepatitis A, B or C, and sexually transmitted infections (STIs): syphilis; gonorrhea; chlamydia; genital herpes; genital condyloma / human papilloma virus; lymphogranuloma venereum proctitis infection; genital mycoplasma; trichomoniasis.

AIDS-defining opportunistic infections, except for pulmonary tuberculosis, were not considered in this analysis. Indeed, we hypothesized that HIV testing would be performed during the diagnostic process surrounding these events.

### Main outcomes

Patients were asked about their encounters with healthcare settings and HIV testing history during the three years prior to HIV diagnosis. For patients belonging to a high-risk group, we determined whether, in the 3-year period prior to HIV diagnosis, they had seen a healthcare provider and, if so, mentioned that they belonged to a high-risk group. For patients presenting with possibly HIV-related conditions, we determined whether they had seen a healthcare provider. Among patients who had seen a healthcare provider from both groups, we determined whether an HIV test had been proposed during the first visit. Medical encounters between patients from high risk groups or with a possibly HIV-related condition and any healthcare provider, which did not lead to a test proposal, were defined as missed opportunities for HIV testing.

When evaluating patient medical history during the past three years, we considered that a patient to whom a test had not been proposed was probably HIV-infected at the time of this contact if: (i) he/she did not report an HIV-negative test after this contact; or (ii) he/she was not diagnosed at an acute HIV infection stage (defined as an incomplete western blot, detectable Agp24, or detectable plasma viral load, with a negative or weakly ELISA, or an interval <3 months between a negative and positive ELISA test [41]).

### Data collection

The face-to-face interview used a standardized questionnaire that anonymously collected information on patient socio-demographic characteristics, risk factors for HIV acquisition, date of the first HIV-positive test, clinical and immunological characteristics at HIV diagnosis, history of HIV testing, contacts with healthcare settings, and clinical events. Possibly HIV-related conditions, occurring during the three years prior to HIV diagnosis, were to be detailed. Patients belonging to high-risk groups and/or presenting with possibly HIV-related conditions and who had seen a healthcare provider were questioned regarding which healthcare facility they had visited and whether or not an HIV test had been proposed.

### Sample size estimate

Age has been found to be associated with missed opportunities for HIV testing in US studies [22,35]. Based on results from studies that have explored delayed HIV testing and hypothesizing that patients with delayed HIV testing are also those with missed opportunities, we expect that 9% of patients with missed opportunities for HIV testing are >50 years of age [14]. For an OR of 2 to be significant when comparing HIV testing missed opportunities in people aged >50 years versus those aged ≤50, assuming an alpha-risk of 5%, a 1-beta risk of 80%, and a bilateral test, 196 people aged >50 and 784 people aged ≤50 had to be enrolled in the study.

### Statistical analysis

Descriptive statistical methods were used to describe the study population and opportunities for HIV testing. Continuous data were compared using the Wilcoxon rank test and categorical data using the Fisher exact test. P-values ≤0.05 (two-tailed) were considered significant.

To differentiate between missed opportunities for testing in patients “belonging to a high risk group” and those “presenting with possibly HIV-related conditions”, we considered only patients without a past history of possible HIV-related conditions when we evaluated opportunities for HIV testing in patients belonging to a risk group.

HIV-related conditions occurring within the 3-month period prior to HIV diagnosis were considered to be directly related to diagnosis. Testing opportunities were therefore evaluated only in patients who had presented HIV-related conditions between 3 years and 3 months prior to HIV diagnosis. When multiple HIV-related conditions had occurred during that period, we included in the analysis only the earliest HIV-related conditions.

Center effect impact on missed opportunities rate for HIV testing was studied by regrouping centers base HIV low of high new diagnosis rate (cut-off: 80 HIV positive tests per million inhabitants [19]). Chi^2^ test was used for the comparisons.

Statistical analyses were performed using SAS 9.2 software (SAS Institute Inc., Cary, NC, USA).

## Results

During the study period, 2,009 newly diagnosed HIV-infected patients sought care in the 69 participating centers throughout France. Overall, 659 (33%) were excluded because they did not fulfill inclusion criteria: 288 had been living in France for <1 year, 95 had had the initial HIV-positive test performed abroad, 148 sought care ≥6 months after HIV diagnosis, 18 were aged ≤18 years, and 110 were excluded for other reasons (mainly because they did not speak French). The study was not proposed to 227 patients mainly because of time pressure and lack of availability of care providers, and 115 more patients refused to participate in the study. Finally, 1,008 patients (50%) were enrolled. The overall 2,009 patients who sought care in study centers were not different from newly diagnosed HIV-infected patients in France during the same period [42] in terms of sex, age, clinical or immunological characteristics at HIV diagnosis (Table 1). However, the 1,008 patients enrolled in the study were more frequently men, older, born in France, diagnosed during acute HIV infection when compared with patients not enrolled.

**Table 1:** Characteristics of patients diagnosed with HIV during the study period

	**Patients included N = 1,008**	**Patients not included N = 1,001**	**p**	**Total N = 2,009**	**2009 French surveillance data**
Sex, number of men (%)	793 (79%)	599 (60%)	<0.0001	1392 (69%)	67%
Born outside of France, n (%)	267 (27%)	418 (52%)	<0.0001	785 (39%)	47%
HIV stage at diagnosis, n (%)					
CD4 <200/mm3	308 (31%)	254 (25%)	0.72	562 (28%)	28%
AIDS	162 (16%)	133 (13%)	0.18	295 (15%)	14%
Acute infection	153 (15%)	87 (9%)	0.0002	240 (12%)	10%
Age at HIV diagnosis, mean (SD)	39.5 (11.6)	35.7 (11.7)	<0.0001	37.6 (11.8)	38.2

Characteristics at HIV diagnosis of patients included or not included in the study from June 2009 to October 2010, versus characteristics of newly HIV-diagnosed patients in 2009 in France (data from French HIV/AIDS surveillance system [42]).

SD: standard deviation.

### *Socio-demographic characteristics at HIV diagnosis* (Table 2)

**Table 2:** Socio-demographic characteristics and HIV testing history of newly HIV-diagnosed patients at diagnosis (n = 1,008)

	**n**^**a**^	**%**
***Sex, men***	793	79
***Age, years***		
18-29	225	22%
30-49	595	59%
≥ 50	188	19%
***Risk factor for HIV***^***b***^		
Intravenous drug user	12	1%
Man who has sex with men	530	53%
Sub-Saharan African immigrant	124	12%
Heterosexual with high risk behavior	155	15%
No identified risk	187	19%
***Educational attainment***		
No certificate	69	7%
High school level (included professional certificate)	542	54%
University level, ≤2 years post high school certificate	146	14%
University level, >2 years post high school certificate	236	23%
***Occupational class***		
Farmers, manual workers	85	8%
Shopkeepers, craftsmen, and office, sales and services employees	479	48%
Professionals, managers and intermediate white-collar workers	242	24%
Unemployed, including retirees and students^c^	191	19%
***Marital status***		
Single	520	52%
Living in couple	358	36%
Divorced or widowed	125	12%
***Children***, ≥1	401	40%
***Health insurance at time of diagnosis***^d^		
Basic health insurance	828	83%
Universal medical coverage^d^	89	9%
Medical aid from state^e^	32	3%
Uninsured or under affiliation	54	5%
Supplementary health insurance	725	72%
***History of HIV testing***		
Never tested	325	32%
HIV tested >3 years	273	27%
HIV tested ≤3 years	407	41%

^a^Depending on variables, missing data were from 0 to 1.5% and are accounted for in the percentages.

^b^Individual class by descending risk: IDU, MSM, Sub-Saharan African migrants, heterosexual with sexual risk, not belonging to a risk group.

^c^32 (3%) retirees; 38 (4%) students.

^d^In France, a universal health care insurance system covers around ¾ of healthcare expenditures. Workers and retirees who contribute to social insurance are covered by the system. Supplemental coverage may be bought by patients from private insurers. The universal medical coverage (complete or supplementary) extends health insurance for all poor legal residents.

^e^Complete health insurance for refugees.

Patient’s median age was 39.5 years; 793 (79%) were males. More than 80% belonged to a high risk group, 12 (1%) were IDU, 530 (53%) were MSM, 155 (15%) were heterosexuals with risky behavior and 124 (12%) were migrants from Sub-Saharan Africa (Table 2). Most had low educational attainment (high school level or less = 61%). Patients were well insured at the time of HIV diagnosis: 772 (77%) had complete health insurance (i.e. basis, plus supplementary health insurance or universal medical coverage); only 54 (5%) were not insured prior to HIV diagnosis.

### History of HIV testing

Except for the test leading to HIV diagnosis, 407 (41%) participants had been tested within the last three years, 273 (27%) had been previously tested but more than three years ago and the remaining 325 (32%) had never been tested for HIV. Among MSM, 94 (18%) had never been tested. In contrast, 45% of sub-Saharan African migrants, 44% of heterosexuals with risky behavior, and 57% of patients who stated not belonging to a risk group had never been tested (p < 0.0001 versus MSM). Among migrants from sub-Saharan Africa, 43 (35%) had a history of HIV testing in France.

### Clinical and immunological characteristics at HIV diagnosis

HIV was diagnosed during acute infection in 153 (15%) patients (Table 1). At diagnosis, 162 (16%) had had an AIDS-defining event and 308 (31%) a CD4 cell count <200 cells/mm^3^. The main circumstances leading to HIV diagnosis were the presence of clinical symptoms for 564 patients (56%), voluntary testing for 264 patients (26%) and health check-up for 176 patients (17%) - including systematic prenatal testing (4%).

### Contact with the healthcare system during the three years prior to HIV diagnosis

Among the 1,008 patients enrolled in the study, 994 (99%) had had ≥1 medical encounter during the 3 years prior to HIV diagnosis: 922 (93%) with a general practitioner and 329 (33%) with an emergency department (Table 3). Up to 90% of patients visited a general practitioner at least once a year.

**Table 3:** Patient’s contacts with the healthcare system during the three years prior to HIV diagnosis

	**n**	**%**
***Contact with a healthcare setting***	994	
General practitioner	922	93%
Medical specialist	649	65%
Hospital	324	33%
Emergency department	329	33%
Other medical department or practitioner	377	38%
***Annual frequency of encounters with general practitioner***		
Never, or did not know	106	11%
Once a year	286	29%
Two to six times a year	511	51%
At least once a month	91	9%
***Time between last medical encounter and HIV diagnosis***		
≤1 year	826	84%
>1 year	93	9%
Did not know	75	7%

### Opportunities for HIV testing in patients belonging to high risk groups (MSM)

**Figure 1:**
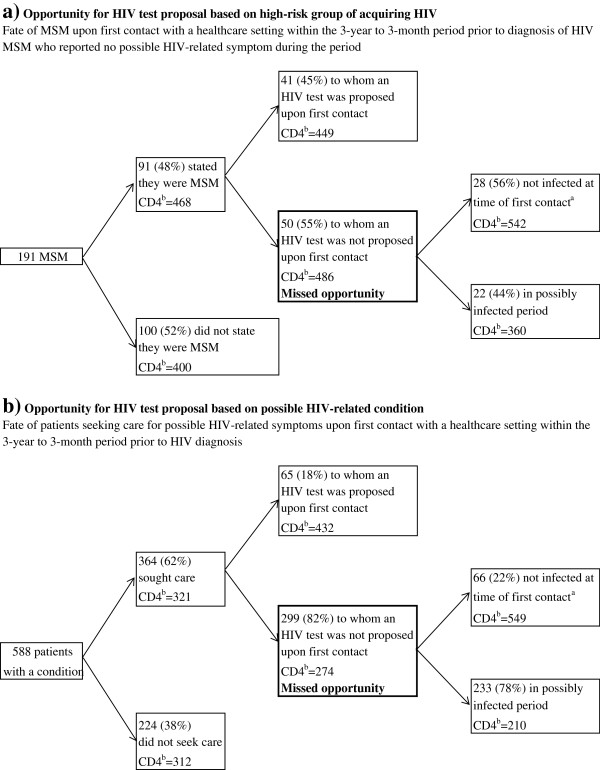
**Opportunity for HIV testing proposal.** Opportunity for HIV testing proposal based on (**a**) group at high risk of acquiring HIV (asymptomatic MSM) and (**b**) any possibly HIV-related condition, among the 994 patients who had contact with the healthcare system in the three years prior to HIV diagnosis. When considering opportunities for HIV testing based on a high-risk group, only patients with no HIV-related conditions in the three years prior to HIV diagnosis were included in the analysis. When considering opportunities for HIV testing based on a HIV-related condition, the first (oldest) symptom reported in the 3-year to 3-month period prior to HIV diagnosis was included in the analysis.^a^Patients were *a posteriori* known not infected for HIV at the first contact when they reported a negative HIV test after this contact or they diagnosed for HIV at acute stage. Others were considered to be possibly infected. ^b^Median CD4 count at diagnosis, cells/μL.

As asymptomatic patients from high-risk groups other than MSM were not in sufficient numbers, description of opportunities for HIV testing in people belonging to high-risk groups were restricted to MSM. Among the 530 MSM enrolled in the study (Figure 1a), 191 reported no possibly-HIV-related conditions and ≥1 contact with a healthcare setting in the past 3-year period. During this contact, 91 (48%) stated to the healthcare provider that they belonged to a high-risk group. Upon first contact, an HIV test was proposed by the healthcare provider to 41 of these 91 MSM (45%). Consequently, 150 MSM of overall 191 asymptomatic MSM (79%) were not tested and had a missed opportunity for HIV testing.

Upon HIV diagnosis, median CD4 count tend to be lower in patients who did not state the healthcare provider that they were MSM compared to those who did so (400 vs. 468 cells/mm^3^, p = 0.08). Among those who stated, median CD4 cell count at diagnosis was not different in MSM whether the test was proposed or not.

### Opportunities for HIV testing in patients with possibly HIV-related conditions

Overall, 364 out of 588 patients who reported a possibly HIV-related conditions during the 3-year to 3-month period prior to HIV diagnosis sought care (Figure 1b). More than half (193/364) sought care for this condition first with a general practitioner, 24% with a medical specialist, 4% with an inpatient service, and 3% with an emergency department.

An HIV test was proposed by the healthcare provider upon first contact to 65 (18%) of these patients. Thus, 299 patients (82%) had a missed opportunity for HIV testing, and 233 (78%) of them were probably infected at the time of this contact (no subsequent HIV test and not diagnosed at an acute HIV infection stage). At HIV diagnosis, median CD4 count was 274 cells/mm^3^ (IQR, 111–507) in patients with a missed opportunity for HIV testing vs. 432 cells/mm^3^ (IQR, 308–621) in those to whom testing was proposed (p < 0.0001).

Results were similar when detailed by HIV-related condition (Table 4). The proportion of HIV testing proposal by the healthcare provider upon the first visit by patients who sought care for a possibly HIV-related condition was low, ranging from 2% for patients with recurrent bacterial infections to 23% for patients with a fever lasting ≥1 month. An HIV test was proposed to half of the patients seeking care for STIs.

**Table 4:** HIV test proposals according to possibly HIV-related conditions

**HIV-related condition**	**N**	**HIV test proposal n(%)**
Recurrent bacterial infection	87	2 (2%)
Generalized lymphadenopathy	46	5 (11%)
Varicella zoster	35	5 (14%)
Unexplained weight loss ≥10%	45	7 (16%)
Diarrhea ≥1 month	36	6 (17%)
Fever ≥1 month	22	5 (23%)
Sexually transmitted infection	101	54 (53%)

Proportion of HIV test proposals upon first visit in patients who sought care for possible HIV-related conditions during the 3-month to 3-year period prior to HIV diagnosis. Main HIV-related conditions are given.

The proportions of missed opportunities for HIV testing in any circumstances were not different between clinical centers when classified following epidemiological characteristics of their belonging regions (data not shown).

## Discussion

This study, which included more than one thousand newly HIV-diagnosed patients in France, demonstrated that 99% of the patients had had at least one medical encounter during the 3-year period prior to HIV diagnosis, and 89% had seen a general practitioner at least once a year during that period. Moreover, results revealed the high proportion of missed opportunities for HIV testing among MSM and those seeking care for possibly HIV-related conditions. Seventy nine percent of overall MSM who visited a healthcare provider did not receive an HIV test proposal. Likewise, HIV testing was not proposed by the healthcare provider to 82% of patients who sought care for possible HIV-related conditions upon first contact.

This study had some strengths and limitations. We enrolled 1,008 newly-HIV-diagnosed patients, who accounted for 16% of new HIV diagnoses in France during the same period [19]. Enrolled patients were not representative of overall newly HIV diagnosed patients because of eligibility criteria. In order to study missed opportunities possibly occurring in France, patients diagnosed overseas or living in France for <1 year were excluded; hence, migrants were underrepresented (27% vs. 47% recorded in 2009 French surveillance data [42]) and MSM were overrepresented (53% vs. 38% recorded in 2009 French surveillance data [42]). Migrants were more likely women, young and being diagnosed for HIV at chronic or advanced stage of HIV infection [42] that explains difference in enrolled and non-enrolled patients. However, characteristics of overall patients were close to those of French surveillance data, and our study sample may be representative of patients who acquired HIV in France.

We evaluated the patient course through the healthcare system in the years prior to HIV diagnosis. That may be subject to recall bias, leading to an underestimation of the number of missed opportunities. To limit this bias, patients diagnosed >6 months previously were excluded, and the period during which retrospective data were collected was restricted to three years. Another limitation may be the misperception of being tested. On one hand, some patients may be tested without knowing the test was performed, without the consent process, leading to overestimate missed opportunities proportion. On another hand, some patients may think having been tested for HIV because a blood analysis was performed during an inpatient stay for example, which may this time, underestimate missed opportunities proportion.

In MSM or patients presenting with possibly HIV-related conditions, we determined whether an HIV test was proposed upon first contact with the healthcare setting. This may underestimate the HIV test proposal rate, since HIV infection may be brought up at the second or a later visit. First, the effect of this bias was reduced by accounting only for visits that had occurred >3 months before HIV diagnosis, ensuring these events did not lead to HIV diagnosis. Next, a high proportion of patients with missed opportunities for HIV testing did not undergo any other HIV test prior to the test that diagnosed the HIV infection.

Only a few asymptomatic people belonging to a high-risk group other than MSM, i.e. sub-Saharan African migrants or heterosexuals with risky sexual behavior, were enrolled in the study and therefore we could not determine missed opportunities for them.

Our results are cause for concern and are in agreement with those reported in the medical literature that overall estimated high proportions of missed opportunities for HIV testing. However, a comparison of our results with other studies on the topic is difficult. Our study is unique in that it investigated the overall patient trajectory though the healthcare system; thus, it evaluated HIV testing strategies based on risk factors and symptoms such as applied currently. Most other studies [22,33-35,43] were conducted in the US at single large urban medical centers with designs based on retrospective medical chart reviews; opportunities for testing that might occur in other facilities could not be collected [22,34,35], thereby underestimating missed opportunities for HIV testing in those studies compared to ours. The population enrolled in other studies was also different from ours in that they focused on specific subgroups; for example, late presenters [33], patients attending STI clinics [36] or immigrants [44]. Moreover, they used different definitions of a missed opportunity. Some authors defined a missed opportunity for testing in newly HIV-diagnosed patients as any medical appointment in the years prior to HIV diagnosis [44]. Others added to this definition the notification of HIV-related risks and/or clinical events (with events varying from one study to another [22,34,36]). Very few authors coupled the presence of HIV-related risks and/or clinical events with the absence of a test proposal by the care provider, which is the definition that we used [35]. As a result, the proportions of missed opportunities for HIV testing in the medical literature range from 20 to 80%.

Our study illustrates the failure to identify persons at high risk of being HIV-positive. In the past three years, >80% of the 191 asymptomatic MSM had seen a healthcare provider. However, only 48% of them informed the provider, either spontaneously or upon questioning, that they belonged to a risk group. Indeed, patients may be wary of the healthcare setting, fearing moral judgment of their behavior, leading to unwillingness to disclose sexual practices to care providers [45]. They may also not consider themselves at risk of HIV infection. This is the main reason why people do not test for HIV, according to a recent systematic literature review summarizing barriers to HIV testing in Europe [21]. In addition, healthcare providers themselves may be ill at ease when dealing with sexuality and behavior risk assessment [46,47].

Even in patients identified as being at high risk of HIV infection, our study illustrates the failure to propose an HIV test: only one test proposed out of two MSM identified. Barriers to HIV testing on the part of the healthcare provider include a lack of training in HIV testing and a lack of self-confidence when proposing it [21]. The consent process and counseling requirements have been reported to be burdensome, particularly for general practitioners [20].

Thus, these findings suggest that risk-factor-based HIV testing is insufficient because its application is insufficient. In this situation, routine HIV testing may be attractive because it does not need risk assessment and follows an opt-out process. However, in US as in France where it is recommended, it is not widely applied because of implementation difficulties, and high costs [30-32,48]. Haukoos *et al.*[49] have recently proposed a score to identify people at risk of being HIV-infected based on eight demographical and behavioral items. Although this score require a minimum of risk assessment and may need validation for French population, it is a starting point to re-think routine HIV testing, and highlight specific populations at risk for HIV to be tested such as men. Routine screening can also be restricted to high prevalence geographical areas.

In patients with STIs, a test was only proposed to one out of two patients although a STI diagnosis is an indication for an HIV test [24,28]. In patients with other HIV-related conditions who sought care, frequencies of missed opportunities were even higher, as high as 98% for those with recurrent bacterial infections. We found that patients who did not receive a test proposal had been diagnosed with significantly lower CD4 cell counts than patients who were proposed a test. This confirms that patients without a test proposal were probably HIV-infected when they consulted and thus had had a loss of chance of being diagnosed and entering into care. It is crucial to bring to healthcare providers this kind of evidence regarding HIV testing for them to improve their knowledge about HIV-related conditions and thus to increase the proportion of patients tested for HIV. An European study has been currently conducted to assess the prevalence of undiagnosed HIV infection in patients with some particular HIV indicator diseases (e.g. mononucleosis-like illness or malignant lymphoma); results show HIV prevalence above 1% in patients with these HIV indicator diseases [50]. This is another argument to convince healthcare providers of the importance of testing proposal for patients with HIV indicator diseases.

## Conclusions

This study illustrates that the rate of missed opportunities for HIV testing remains unacceptable and highlights the low efficiency of risk-factor- and symptom-based HIV testing as it is currently applied. We must therefore reinforce and implement new HIV screening strategies. Testing initiated by the healthcare provider should be promoted. Because risk-factor based testing is not sufficient, routine screening should be considered in particular in high prevalence geographical areas and/or specific populations. In our study, most persons had contact with healthcare facilities and particularly general practitioners who should play an important role in future HIV testing strategies. But we must train physicians to better recognize HIV-related conditions and develop tools such as behavioral and clinical instruments to better identify those at high risk of HIV infection in whom HIV testing should be regularly performed. HIV testing has become a major tool and the basis of the new prevention strategies such as the Test and Treat strategy; it should be promoted urgently.

## Ethical statement

The study received approval from two French data protection authorities (CCTIRS, Comité Consultatif sur le Traitement de l’Information en matière de Recherche dans le domaine de la Santé, and CNIL, Commission Nationale de l’Informatique et des Libertés) and did not requite formal ethical approval according to French regulations. All patients signed an informed consent form before taking part to the study.

## Abbreviations

CCTIRS: Comité Consultatif sur le Traitement de l’Information en matière de Recherche dans le domaine de la Santé; CNIL: Commission Nationale de l’Informatique et des Libertés; ELISA: Enzyme-linked immunosorbent assay; HIV: Human immunodeficiency virus; IDU: Injected drug user; MSM: Man who have sex with man; UK: United Kingdom; US: United States.

## Competing interest

No authors declare association that might pose a conflict of interest with the submitted work. However, LC received travel grants, participated in advisory boards, or received fares for public presentations from Gilead, Boehringer Ingelheim, Janssen-Tibotec, BMS, ViiV, MSD, and Pfizer. SDB received grants from Roche, Janssen-Cilag and Schering-Plough, and received consultancy honoraria from Merck and GlaxoSmithKline. YY received travel grants, honoraria for presentations at workshops and consultancy honoraria from Abbott, Bristol-Myers Squibb, Gilead, Merck, Roche, Tibotec and ViiV Healthcare.

## Authors’ contribution

All authors contributed to the study. KC, SLV, SDB, CS and YY had the idea for the study. All authors substantially contributed to the conception, design and feasibility of the study. KC wrote the protocol and questionnaires used in the study. LC, KL and YY tested the questionnaires. KC coordinated the study. PB was responsible for management of data. KC and AC performed statistical analyses and presented results. All authors participated in interpretation of results. KC and YY drafted the article. All authors critically revised the manuscript for important intellectual content and approved the final version.

## Pre-publication history

The pre-publication history for this paper can be accessed here:

http://www.biomedcentral.com/1471-2334/13/200/prepub
